# New enlightenment on the regulatory effects of acids and phenolic compounds in wood vinegar, a by-product of biomass pyrolysis, on tomato production

**DOI:** 10.3389/fmicb.2025.1538998

**Published:** 2025-07-16

**Authors:** Hongyin Zhou, Naiming Zhang, Liyuan Mu, Liu Gao, Li Bao, Caixian Tang

**Affiliations:** ^1^College of Plant Protection, Yunnan Agricultural University, Kunming, China; ^2^Yunnan Key Laboratory of Gastrodia and Fungi Symbiotic Biology, Zhaotong University, Zhaotong, China; ^3^College of Resources and Environment, Yunnan Agricultural University, Kunming, China; ^4^Centre for AgriBioscience, La Trobe University, Melbourne, VIC, Australia

**Keywords:** wood vinegar, acids, phenols, soil properties, microbial environment, regulatory effects

## Abstract

Wood vinegar (WV), a biomass pyrolysis by-product, is widely used in agriculture because of the complexity and abundance of its bioactive substances. However, the specific mechanisms underlying plant growth regulation by acids and phenolic compounds, accounting for the largest proportion of their constituents, remain unclear. Therefore, the main acids (N-ethylglycine, Lactic, and 2-pyridinecarboxylic acids) and phenols (Catechol and Guaiacol) were selected to understand their effects on soil properties, microbial communities, and tomato growth in a pot experiment. Results showed that individual applications of acids and phenolics significantly enhanced nutrient availability (e.g., soil AP, AK, and AN), promoted tomato growth (e.g., PH, SD, AB), and regulated endogenous hormone levels (upregulating auxin (IAA), gibberellin (GA3 ), and cytokinin (CTK); downregulating abscisic acid (ABA). Among them, N-ethylglycine and catechol exhibited the most pronounced effects. However, their mixture (acid-phenolic combination) attenuated growth-promoting effects and hormonal regulation, accompanied by reduced bacterial community richness (ACE and Chao indices) and operational taxonomic units (OTUs) compared to single treatments. The dominant bacteria in the treatments with N-ethylglycine, Catechol, and their combination were *Pedobacter*, *Pseudoxanthomonas*, and *TM7a*, respectively, whereas the dominant fungi were *Olpidiaster*, *Borealophlyctis*, and *Spizellomyces*, respectively. Network-based analysis showed that *Pseudoxanthomonas* was negatively correlated with *Pedobacter*, and *Olpidiaster* was positively correlated with *Spizellomyces*. These findings deepened our understanding of the effects of acids and phenolics in WV on endogenous hormone levels, soil chemical characteristics, microbial diversity, and metabolic processes in tomato, and revealed the mechanisms of regulatory effects of WV components on crop growth.

## Graphical Abstract

The regulatory mechanism of 2—pyridinecarboxylic acids and Catechol in wood vinegar, a by—product of biomass pyrolysis, on tomato growth. 
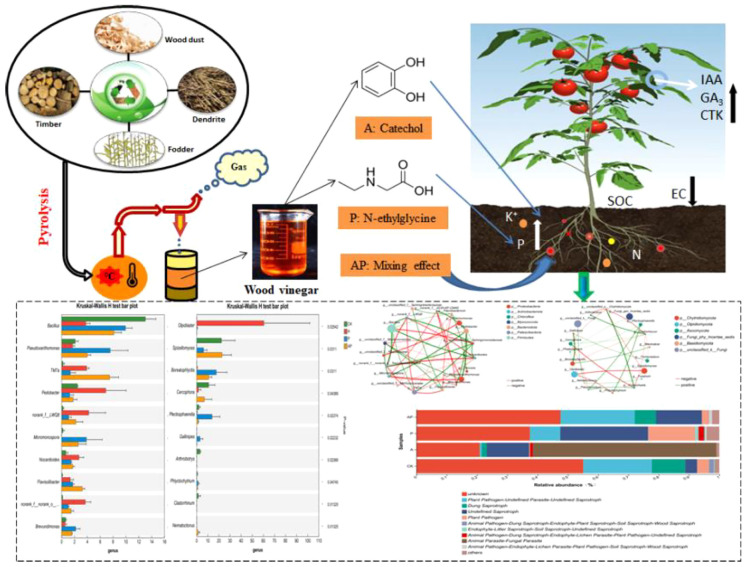

## 1 Introduction

The variety and quantity of agricultural and forestry wastes generated annually are enormous. Global agriculture, forestry, and industry produce 200 billion tons of lignocellulosic biomass annually (Dahmen et al., 2019; Zhou et al., 2024). In the current context of climate change, energy shortages, environmental pollution, food crises, and limited sustainable agricultural development, biomass charcoal, based on the resource utilization of agricultural and forestry wastes, has become a globally recognized renewable energy source because of its carbon sequestration and emission reduction functions, and its advantages of achieving carbon neutrality and low pollution in energy production (Zhou et al., 2024). Pyrolysis, a widely accepted technology for bioenergy development, is effective in reducing the environmental problems associated with conventional biomass combustion (Zhang et al., 2019). With the increasing demand for biochar, the production of wood vinegar (WV) along with biochar has also increased, whereas WV as a by-product will produce new pollution if it is not disposed of properly; therefore, the exploitation of WV has become imperative in recent years (Li et al., 2019; Zhang et al., 2019).

WV is a reddish-brown acidic liquid with a special smoky aroma obtained by the condensation of flue gases produced during the pyrolysis of biomass from agricultural and forestry waste, such as straw, woodchips, and twigs. WV has a pH of approximately 3 (Mathew and Zakaria, 2015), and a complex and diverse composition, containing abundant organic compounds such as acids, phenols, ketones, aldehydes, alcohols, esters, ethers, and furans (Grewal et al., 2018). As a new type of green and natural material, WV has various uses in agriculture, industry, and environmental protection. For example, WV can increase soil pH, improve soil characteristics, promote soil microbial activity, promote plant growth, and enhance crop disease resistance (Mungkunkamchao et al., 2013; Zhou et al., 2023); it can inhibit germs and repel pests (Hu et al., 2022; Wang et al., 2015; Xue et al., 2022); it can be used as an antiseptic and de-icer (Song et al., 2022; Xue et al., 2023); and can reduce NH_3_ and greenhouse gases and play a role in air purification (Quan et al., 2021).

WV is the most widely used plant growth regulator in agriculture (Zhou et al., 2023). WV has a highly efficient growth-promoting effect. Simma et al. (2017) showed that the WV treatment significantly increased the seed germination rate and growth of rice plants. Mungkunkamchao et al. (2013) showed that spraying with WV significantly increased crop yield, which may be because WV is rich in organic components, including organic acids and various forms of phenols, alcohols, and esters. Lu et al. (2019) demonstrated that WV improves the seed germination rate and promotes plant growth, and its regulatory properties are related to its acid and phenol contents.

Although the application potential of WV is remarkable, there are still many deficiencies in existing research. Most of the existing studies use crude WV, whose components are significantly affected by raw materials and pyrolysis processes, and it contains harmful substances such as tar and formaldehyde (Mungkunkamchao et al., 2013). At the same time, most studies only analyze the overall effect of WV without isolating and identifying specific active components. Although studies have pointed out that it exerts its effects by regulating plant hormones (such as gibberellin and cytokinin) and antioxidant enzyme activities (such as SOD and POD) (Lu et al., 2019), the synergistic effect mechanism of acids and phenols remains unclear. In addition, the role of soil microorganisms in the action of WV is still unclear. Most of the existing studies focus on a single microbial group (such as bacteria), lacking a systematic analysis of soil microbial networks and functional genes. In response to these issues, this study aims to promote the efficient utilization of WV through the following innovative strategies. (1) Conduct a comprehensive component analysis of WV using GC-MS, and combine it with bioactivity screening to clarify the contents and proportions of major acidic components (such as N-ethylglycine and Lactic acid) and phenolic components (such as Catechol and Guaiacol). Compared with existing studies, this study for the first time directly correlates the chemical composition of WV with its biological effects, providing a material basis for subsequent mechanism research. (2) Through pot experiments, analyze the effects of acid-phenol compounds on tomato endogenous hormones (such as IAA, ABA) and the antioxidant system (such as MDA, SOD), and reveal their growth-promoting and stress-resistant mechanisms. Combine high-throughput sequencing to analyze the regulatory effects of acid-phenol substances on the soil microbial community structure, functional genes, and metabolites. Verify the synergistic effect of acid-phenol compounds through single-component and mixed-component comparison experiments.

The innovation of this study lies in systematically revealing the synergistic mechanism of acid-phenol compounds in WV for the first time, filling the research gap in the plant-soil-microbe interaction network. Promote the transformation of WV from a “by-product” to an “efficient plant growth regulator,” contributing to the circular utilization of biomass resources and the green development of agriculture. The results of this study are expected to provide a new perspective for the functional development of WV, and at the same time provide technical support for the resource utilization of agricultural and forestry waste and the realization of the carbon neutrality goal.

## 2 Materials and methods

### 2.1 Test material

The tomato seedlings (*Solanum lycopersicum* L. cv. Hairy Pink 802) were purchased from Shandong Shouguang Kaijiate Agricultural Science and Technology Co., Ltd. China.

The test soil was red loam collected from the Back Mountain Farm of Yunnan Agricultural University and was thoroughly mixed with rotted sheep manure at a mass ratio of 6:2. The soil-manure mix was then autoclaved at 120°C overnight three times (45 min each). The soil had pH 7.22, organic matter (OM) content 16.9 g/kg, alkali-hydrolyzed nitrogen (AN) 40.1 mg/kg, available phosphorus (AP) 20.2 mg/kg, available potassium (AK) 73.2 mg/kg.

The tested WV is refined wood vinegar, which is prepared by mixing typical wastes from three major fields, namely crop corn straw, forestry waste Chinese fir wood chips, and horticultural pruned grape branches, in a mass ratio of 1:1:1 (It is prepared by Yunnan Kunyu Environmental Development Co., Ltd. China. Process: at medium temperature, < 500°C, with a heating rate of 10^4^ K⋅s^–1^, rapid circulation of hot steam to obtain crude WV. The crude WV is allowed to stand for 3 months, and the clear liquid in the middle layer is sucked by siphon method. 5% activated carbon is added, and the mixture is ultrasonically oscillated for 10 min, then allowed to stand for 36 h, and filtered by suction three times to obtain refined WV.). In the early stage of the experiment, multiple rounds of pot screening experiments have been carried out, verifying that the treatment with the WV diluted 200–400 times has a good growth—promoting effect on the growth and development of tomato plants. The chemical components of the wood vinegar were analyzed by Agilent 7890 gas chromatography-mass spectrometry (GC-MS). Compounds were identified by comparing the retention time and the mass spectra with the data in the mass spectrometry library (NIST). The content of the compounds was determined by the relative peak area (Ma et al., 2013). The content of the main chemical components is shown in Supplementary Table S1.

In this experiment, the organic acid components (N-glycylglycine, Lactic acid, and 2-pyridinecarboxylic acid) and phenolic organic components (Catechol and Guaiacol) with the highest content in WV were selected as the test materials (Organic components were purchased from Shanghai Yien Chemical Technology Co., Ltd. China.). The pot experiment was carried out to simulate and verify the growth-promoting and regulating effects of acid and phenolic substances in WV with different dilution concentrations on tomatoes. Configuration of single chemical component simulation solution: accurately weigh a certain amount of a single acid and phenolic substances, diluted with different multiples of sterile water, and formulated into different concentrations of a single component simulation solution to be used; weigh three types of acids and two types of phenols mixed with each other, diluted with different multiples of sterile water, and formulated into different concentrations of acid-phenol mixture simulation solution.

### 2.2 Experimental design and sample collection

The experiment was conducted from March 2023 in an experimental greenhouse at Yunnan Agricultural University, Kunming, Yunnan Province. The plants were planted in pots with a diameter of 10 cm at the bottom, height of 12.5 cm, and soil weight of 3 kg per pot. They were planted on March 29, 2024, with three strong seedlings with uniform growth in each pot. The experiment was set up with 36 treatments using distilled water as the control; the experimental design is shown in Table 1. Three pots were planted for each treatment and 108 pots were planted. All treatments involved root irrigation on the 5th (five-leaf stage), 12th, 19th, and 26th days after planting, and the irrigation amount was 200mL/pot. All other management techniques were performed according to conventional cultivation practices. The biomass survey was conducted on day 33, inter-root soil samples were then collected, and 20 g of fresh soil samples were taken from each pot and immediately stored in a freezer at −80°C for backup. The remaining samples were air dried to assess their chemical properties. The statistical results of the biomorphology and soil chemical properties for all treatments are shown in Supplementary Tables S2, S3, respectively.

**TABLE 1:** Details of experimental treatments.

Wood vinegar	Treatment	Treatment code	Concentration
	Comparison	CK	Distilled water
Acids	N-ethylglycine acid	A1	Each treatment was prepared in sterile water at concentrations of 30, 60, and 90 mg/L simulants.
	lactic acid	A2	
	2-pyridinecarboxylic acid	A3	
Phenols	Catechol	P1	
	Guaiacol	P2	
Acids + phenols	N-ethylglycine acid + Catechol	AP1	Acids and phenols of the same concentration were mixed with each other to form 30, 60, and 90 mg/L simulants.
	N-ethylglycine acid + guaiacol	AP2	
	Lactic acid + catechol	AP3	
	Lactic acid + guaiacol	AP4	
	2-pyridinecarboxylic acid + catechol	AP5	
	2-pyridinecarboxylic acid + guaiacol	AP6	

### 2.3 Determination of biological traits and endogenous hormones

Biological traits plant height (PH), stem diameter (SD), and leaf area (LA) were determined as in Zhou et al. (2024); underground biomass (UB) and above-ground biomass (AB) samples were dried to a constant weight and then their dry weights were determined using a balance. The endogenous hormones indole acetic acid (IAA), abscisic acid (ABA), gibberellin (GA_3_) and cytokinin (CTK) were extracted from the samples by isopropanol/water/hydrochloric acid extraction, and detected by an Agilent 1290 high performance liquid chromatograph (HPLC) in tandem with a Qtrap 6500 mass spectrometer of AB, and the determination was performed by high performance liquid chromatography coupled with electrospray tandem mass spectrometry (ESI- HPLC-MS/MS).

### 2.4 Determination of chemical properties of soil

The soil pH was determined using a compound electrode at a soil-water ratio of 10:25 (w/v). Soil electrical conductivity (EC) was assessed using a conductance electrode at a ratio of 1:5 (w/v). The soil was heated and oxidized using potassium dichromate and sulfuric acid, after which a ferrous sulfate standard solution was used for titration to determine the OM content. Soil AN was alkali-hydrolyzed with sodium hydroxide and boric acid and filtered. Ammonium bicarbonate was added to the filtrate and the absorbance was determined using an ultraviolet-visible spectrophotometer. Soil AP was extracted with an ammonium fluoride solution, mixed with a molybdenum antimony anti-coloring agent, and then measured using a spectrophotometer. The AK content in the soil was extracted using a neutral ammonium acetate solution and measured using a flame photometer.

### 2.5 Microbial high-throughput sequencing

DNA was extracted from soil samples following the protocol provided by the E.Z.N.A. Soil DNA Kit. The purity of the extracted DNA was assessed by 1% agarose gel electrophoresis. After PCR amplification, preliminary quantification results were obtained through electrophoresis and further quantified using the QuantiFluor™-ST Blue Fluorescence Quantification System (Promega, WI, United States), and then high-throughput sequencing was performed based on the PE: 2 × 300 bp sequencing strategy using the IlluminaMiseq platform. The hypervariable region V3–V4 of the bacterial 16SrRNA gene was amplified with primer pairs 338F (5′-ACTCCTACGGAGGCAGCAG-3′) and 806R (5′-GGACTACHVGGGTWTCTAAT-3′). The hypervariable regions of fungal genes, ITS1F-ITS2R, were amplified with primers for ITS1F (5′-CTTGGTCATTTAGAGGAAGTAA-3′) and ITS12R (5′-GCTGCGTTCTTCATCGATGC-3′). After the PE reads were split into samples, the bipartite reads were subjected to quality control and filtering based on the sequencing quality. They were simultaneously assembled according to their overlapping relationships to produce optimized data following quality-controlled splicing. Finally, the optimized data were processed using the sequence noise reduction method (DADA2/Deblur) to obtain AmpliconSequenceVariant representative sequence and abundance information. Extraction, amplification, library construction, sequencing, and data analysis of microbial DNA from the soil samples were performed by Shanghai Meiji Biomedical Technology Co. (China).

### 2.6 Statistical analysis

SPSS v20.0 software (SPSS Inc., Chicago, IL, United States) and one-way analysis of variance (ANOVA) were used to analyze the tomato biological traits, endogenous hormones, soil chemical properties and soil microbial α-diversity of tomato plants treated with 90 mg/L N-ethylglycine acid (A), 90 mg/L Catechol (P), mixed treatment (AP) and control treatment. Origin 2021 software was used for mapping. The figure and table present the mean and SD values, with *P* < 0.05 deemed significant. Species diversity in each sample was measured using the α-diversity index via QIIME2 software. Principal coordinate analysis was used to assess the diversity among samples. Redundancy analysis (RDA) was performed in R (R Foundation for Statistical Computing, Vienna, Austria) using the “vegan” package to explore microbiome differences across various factors. Sequencing data were analyzed using the MajorBio online platform.^1^ To compare species composition, functional gene predictions, and functional differences between groups, PICRUSt software and the Fungus Functional Guild (FUNGuild) tool were used. Visualizations were created using the R software (V4.0.3).

## 3 Results and discussion

### 3.1 Tomato growth and endogenous hormone levels

**FIGURE 1:**
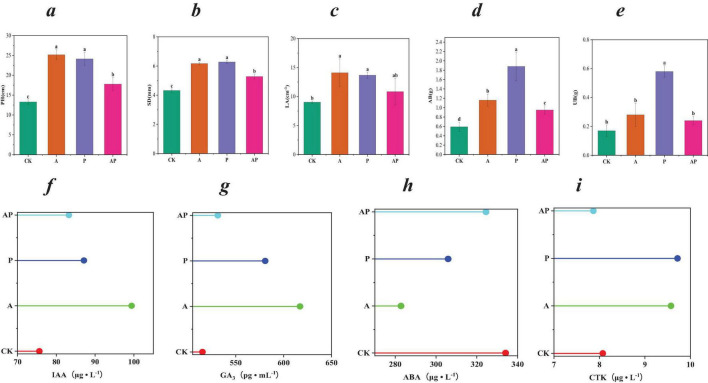
Biological traits and endogenous hormones. **(a)** Plant height (PH); **(b)** stem diameter(SD); **(c)** leaf area (LA); **(d)** aboveground dry weight (AB); **(e)** underground dry weight (UB); **(f)** growth hormone indoleacetic acid (IAA); **(g)** gibberellin 3 (GA3); **(h)** abscisic acid (ABA); **(i)** cytokinin (CTK). CK, control treatment; A, 90 mg/L N-ethylglycine acid; P, 90 mg/L Catechol; and AP, mixed treatment. The values in the table are average ± SD (*n* = 9). Different lowercase letters indicate significant difference among treatments at *P* < 0.05.

The composition of WV is very complex, but studies have shown that its main components are acids and phenolics (Supplementary Table S2), both of which have high biological activities to promote plant growth (Benzon and Lee, 2016; Zhang et al., 2023). In this study, we showed that the major acids (N-ethylglycine acid, Lactic acid, and 2-pyridinecarboxylic acid) and phenols (Catechol and Guaiacol) in WV could promote tomato growth and development and all had a more pronounced enhancement effect with increasing concentration (Supplementary Table S2). In addition, this study found that when acid and phenolic substances were mixed, its growth-promoting effect on tomato was lower than that of acid and phenolic substances alone (Supplementary Table S2). One-way analysis of variance showed that N-ethylglycine acid (A) treatment, catechol (P) treatment and their mixture treatment (AP) significantly increased the PH and SD of tomato. Compared with CK, significantly increased PH by 89.68, 81.84, and 33.91%, respectively, and significantly increased SD by 42.82, 45.37, and 22.22%. In addition, the three treatments also significantly increased tomato LA and tomato AB and UB. The promotional effects of A, P and AP treatments on the growth and development of tomato plants were as follows: A = P>AP (Figures 1a–e). Acids, phenols and other organic compounds in WVare typical allelochemicals that regulate plant growth by affecting ion concentration, respiratory metabolism, hormone balance and protein synthesis (Gulzar et al., 2016; Latif et al., 2017). Yu et al. (2013) pointed out that organic compounds such as phenolic acids have an important effect on plant growth. The H provided by acidic substances destroys the physical interaction between xyloglucan and other compounds in the cell wall, which helps H enter cells and increase organic acids to cause intercellular acidification, thereby increasing plant vitality and promoting plant growth. Phenolic compounds in wood vinegar, especially diphenols and polyphenols, can stimulate the increase of IAA, GA and various enzyme activities at low concentrations and promote plant growth (Zhu et al., 2021). This study also showed that both A and P treatments significantly up-regulated the contents of IAA, GA3 and CTK, and down-regulated the content of ABA in tomato. In addition, compared with the single application treatment, the response intensity of the mixed treatment (AP) to tomato endogenous hormone levels was lower (Figures 1f–i).

Therefore, the acids (N-ethylglycine acid) and phenols (Catechol) in WV had a good growth-promoting effect on tomato growth, while the mixed application of acids (N-ethylglycine acid) and phenols (Catechol) in WV had a certain antagonistic effect on the growth and development of tomato plants. The antagonism of acids and phenols may include binding or non-activation caused by chemical reactions and competitive adsorption or binding (Lv et al., 2016).

### 3.2 Soil chemical properties

The chemical properties of soil have important effects on plant growth (Liang et al., 2024). The acids and phenols in WV had different effects on the chemical properties of the soil (Supplementary Table S3). A one-way ANOVA showed that N-ethylglycine acid and Catechol in WV significantly affected the chemical properties of tomato soil (Table 2). Compared with CK, the A treatment significantly decreased soil pH, whereas the P treatment significantly increased soil pH. Both the A and P treatments significantly decreased soil EC and significantly increased soil OM content. Differences in the effects of AP treatments on soil pH, EC, and OM content were not significant compared to CK. In addition, all treatments significantly increased soil AP, AK, and AN contents compared with CK. The enhancement effects were in the order of P > A > AP. Zhou et al. (2022) showed that various acid phenolics provided by WV were effective in increasing soil TC, TN, AC, and AN content and that the acids (organic acids) in WV significantly reduced soil pH, particularly at high concentrations. One consequence of this pH effect was the reduction in the immobilization of some nutrients in the interroot soil, such as by promoting the release of N and P (Sharma et al., 2016), which increased nutrient effectiveness and promoted plant growth, which is consistent with the results of the present study.

**TABLE 2:** Chemical properties.

Treatments	pH	EC(μS/cm)	OM(g/kg)	AP(mg/kg)	AK(mg/kg)	AN(mg/kg)
CK	6.89 ± 0.02b	2798 ± 87a	27.5 ± 2.0c	6.79 ± 0.87d	391 ± 67.42c	231.23 ± 0.53d
A	6.73 ± 0.10c	656 ± 128c	53.1 ± 4.8a	23.93 ± 0.88b	1066 ± 7.21a	276.15 ± 10.50b
P	7.06 ± 0.04a	1512 ± 155b	42.5 ± 1.8b	30.40 ± 3.02a	1144 ± 60.48a	321.65 ± 3.50a
AP	6.86 ± 0.05b	2616 ± 101a	33.2 ± 2.9c	19.39 ± 0.76c	654 ± 35.12b	255.15 ± 7.00c

OM, organic matter; AN, alkali-hydrolyzed nitrogen; AP, available phosphorus; and AK, available potassium. CK, control treatment; A, 90 mg/L N-ethylglycine acid; P, 90 mg/L Catechol; and AP, mixed treatment. The values in the table are average ± SD (*n* = 3). Different lowercase letters indicate significant difference among treatments at *P* < 0.05.

### 3.3 Microbial diversity analysis

**FIGURE 2:**
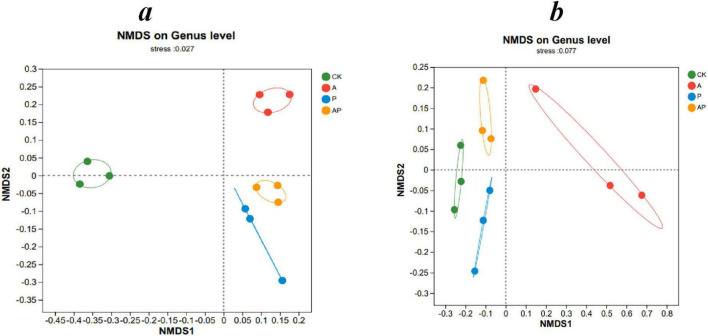
Beta-diversity analysis, non-metric multidimensional scaling analysis (NMDS). **(a)** Bacteria, **(b)** fungi. CK, control treatment; A, 90 mg/LN-ethylglycine acid; P, 90 mg/L Catechol; and AP, mixed treatment. The values are means ± SD (*n* = 3). Different lowercase letters indicate significant difference among treatments at *P* < 0.05. Points of different colors represent samples of different groups. The horizontal and vertical coordinates indicate relative distance, which has no practical significance. stress: test the strength of NMDS analysis results.

The sequencing coverage of bacteria and fungi in tomato interroot soil of all treatments reached > 99% (Table 3), indicating that the sequencing results of all treatments could reflect their real situation. Both acids (A) and phenolics (P) in the WV altered the diversity and abundance of tomato interroot soil. Compared with the control, the A, P, and AP treatments significantly enhanced the ACE and Chao indices of bacteria, whereas there was no significant difference in the ACE and Chao indices of fungi, which indicated that the A, P, and AP treatments could enhance the abundance of soil bacteria. AP treatment significantly reduced the ACE and Chao indices of bacteria compared with A and P treatments, indicating that AP treatment had a certain inhibitory antagonistic effect on the abundance of soil bacteria. Among all treatments, the P treatment had the most significant effect on the bacterial ACE and Chao indices, whereas the A treatment had the most significant effect on the soil bacterial and fungal Simpson indices. Zhang et al. (2019) showed that WV caused more significant changes in microbial species diversity than other treatments. This conclusion was confirmed by the fact that phenolics (Catechol) in WV obtained in this study had the most significant effect on enhancing the abundance of soil bacteria, whereas acid (N-ethylglycine acid) had the most significant effect on enhancing the diversity of soil bacteria and fungi. Non-metric multidimensional scaling analysis (NMDS) was used to compare the dissimilarity of bacterial and fungal community composition in different treatments. There were significant differences in the composition of bacterial and fungal communities between different treatments (Figures 2a,b).

**TABLE 3:** Alpha diversity estimators.

Microorganisms	Treatments	ACE index	Chao index	Simpson index	Coverage rate%
Bacteria	CK	1139 ± 134b	1124 ± 124b	0.013 ± 0.003a	99.82
	A	1269 ± 123ab	1243 ± 121ab	0.015 ± 0.005a	99.62
	P	1561 ± 314a	1540 ± 320a	0.006 ± 0.002b	99.6
	AP	1203 ± 131ab	1187 ± 119ab	0.010 ± 0.001ab	99.74
Fungi	CK	95 ± 22a	95 ± 21a	0.20 ± 0.03ab	99.99
	A	80 ± 23a	79 ± 23a	0.51 ± 0.31a	99.99
	P	117 ± 58a	117 ± 57a	0.11 ± 0.02b	100
	AP	126 ± 41a	126 ± 41a	0.22 ± 0.12ab	99.99

CK, control treatment; A, 90 mg/L N-ethylglycine acid; P, 90 mg/L Catechol; and AP, mixed treatment. The values in the table are average ± SD (*n=3*). Different lowercase letters indicate significant difference among treatments at *P* < 0.05.

### 3.4 Difference analysis of rhizosphere soil microbial community

Venn diagram analysis showed that there were 2,648, 2,677, 3,495, and 2,409 bacterial operational taxonomic units (OTUs) in the CK, A, P, and AP treatments, respectively, and the numbers of unique bacterial OTUs were 2,146, 1,952, 2,555, and 1,396, respectively. There were 188, 150, 264, and 276 fungal OTUs, respectively. The numbers of unique fungal OTUs were 140, 98, 205, and 215, respectively. The number of soil bacterial OTU in treatments A and P was higher than in the control, whereas the number of soil bacterial OTU in the AP treatment was lower than in the control.

**FIGURE 3:**
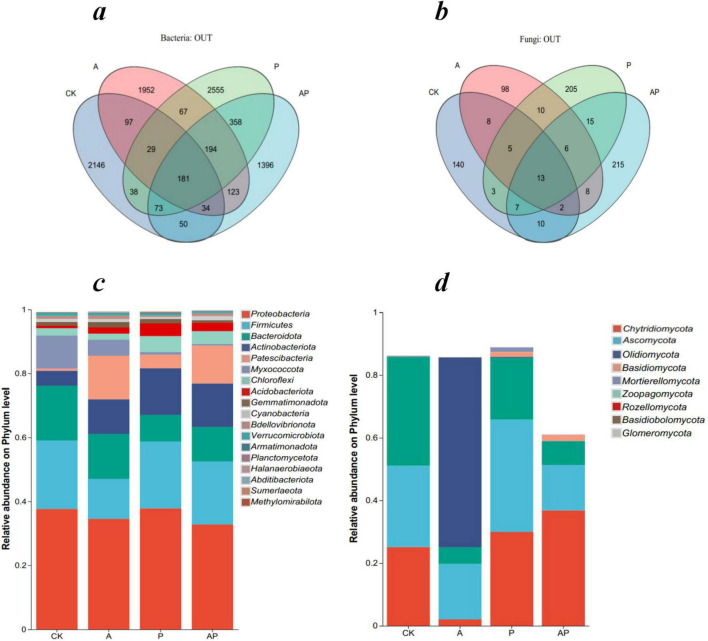
Comparative analysis of microbial community composition under different treatments. CK, control treatment; A, 90 mg/L N-ethylglycine acid; P, 90 mg/L Catechol; and AP, mixed treatment. **(a)** Venn diagram of soil bacterial OTUs, **(b)** Venn diagram of soil fungal OTUs, **(c)** the relative abundances of bacterial phyla, and **(d)** the relative abundances of fungal phyla.

Comparative analysis of the relative abundances of soil fungi and bacterial phyla under different treatments revealed that the community composition varied significantly among treatments, with the top five bacterial phyla of the soil community being *Proteobacteria*, *Firmicutes*, *Bacteroidota*, *Actinobacteriota*, and *Patescibacteria*, which varied in proportion (Figure 3). All treatments significantly increased the relative abundance of *Actinobacteriota* and *Patescibacteria* communities compared to CK, with the AP treatment increasing the relative abundance of *Actinobacteriota* communities less than the A treatment and increasing the relative abundance of *Patescibacteria* communities less effectively than the P treatment The increase in the relative abundance of *Patescibacteria* was lower than that of P treatment. The effect of AP treatment on the relative abundance of *Actinobacteriota* was lower than that of treatment A, and the effect on the relative abundance of Patescibacteria was lower than that of treatment P. The results showed that the AP treatment had an antagonistic effect on the increase in the relative abundance of bacterial communities.

The top five fungal phyla in the soil community were *Chytridiomycota*, *Ascomycota*, *Olpidiomycota*, *Basidiomycota*, and Mortierellomycota, with varying proportions (Figure 3). The A treatment’s *Chytridiomycota* and *Ascomycota* communities were lower in relative abundance than that of CK, and *Chytridiomycota* and *Ascomycotacommunities* were higher in relative abundance than CK in the P treatment, whereas AP treatment significantly increased the relative abundance of the *Chytridiomycota* community and significantly decreased the relative abundance of the *Ascomycota* community. These findings also validate the inference of previous studies that major organic components, such as acids, phenols, and ketones, in WV affect soil microbial community structure and characteristics (Gómez-Acata et al., 2017; Leong et al., 2011).

### 3.5 Analysis of microbial species differences

**FIGURE 4:**
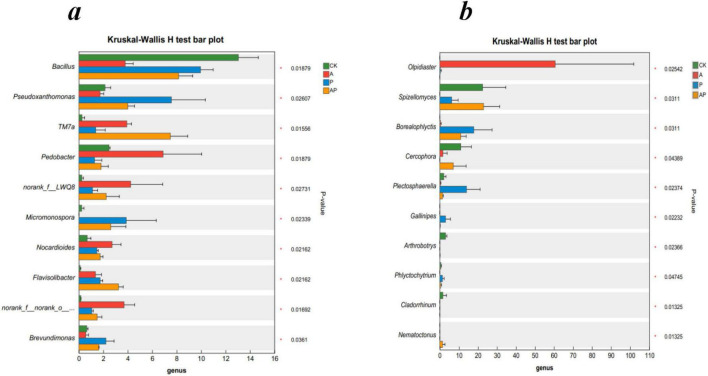
The relative abundances of bacteria **(a)** and fungi **(b)** at the genus levels. CK, control treatment; A, 90 mg/LN-ethylglycine acid; P, 90 mg/L Catechol; and AP, mixed treatment. Error bars are inferential error bar. The *P-*values of treatment differences are shown.

Figure 4 shows the differences in the mean relative abundances of the same species at the genus level for soil bacteria and fungi between different groups, visualizing the significance of the differences between the same species across multiple groups (Figures 4a,b). The results showed that for soil bacteria, the relative abundance of *Pedobacter* in the A treatment was the most significant compared to the other treatments, with a relative abundance of 6.89%; the relative abundance of *Pseudoxanthomonas* in the P treatment was the most significant compared to the other treatments, with a relative abundance of 7.57%. The relative abundance of *TM7a* in the AP treatment was significantly higher than that in the other treatments, with a relative abundance of 7.49%, indicating that the relative abundance of the species in the A treatment was significantly higher than that in the other treatments, indicating that *Pedobacter* in treatment A and *Pseudoxanthomonas* in treatment P were the dominant microbial species for tomato growth promotion. *Pedobacter* is a genus of *Bacteroidota*, a gram-negative bacterium capable of using heparin as the sole carbon and nitrogen source, and *Pedobacter* encodes many diverse resistance mechanisms with intrinsic resistance to environmental antibiotics, making it an environmental superbug (Viana et al., 2018). *Pseudoxanthomona*, a genus of *Proteobacteria*, plays a crucial role in soil nutrient metabolism (Fu et al., 2019; Guo et al., 2020; Ibrahim et al., 2020). Bacterial species *TM7a* was the dominant species in the mixed A and P treatments.

For fungi, among all the treatments, the highest relative abundance of *Olpidiaster* was found in treatment A with 60.60%. *Borealophlyctis* and *Plectosphaerella* species were found in treatment P with 17.76 and 13.93%, respectively, whereas the relative abundance of AP and control treatments for the *Spizellomyces* species showed non-significant differences in relative abundance of 22.88 and 22.39%, respectively, but were significantly higher than that in the A and P treatments. *Spizellomyces* is a taxon of fungi that includes plant pathogens (Chen et al., 2018).

### 3.6 Relationship between microbial community structure and soil properties

**FIGURE 5:**
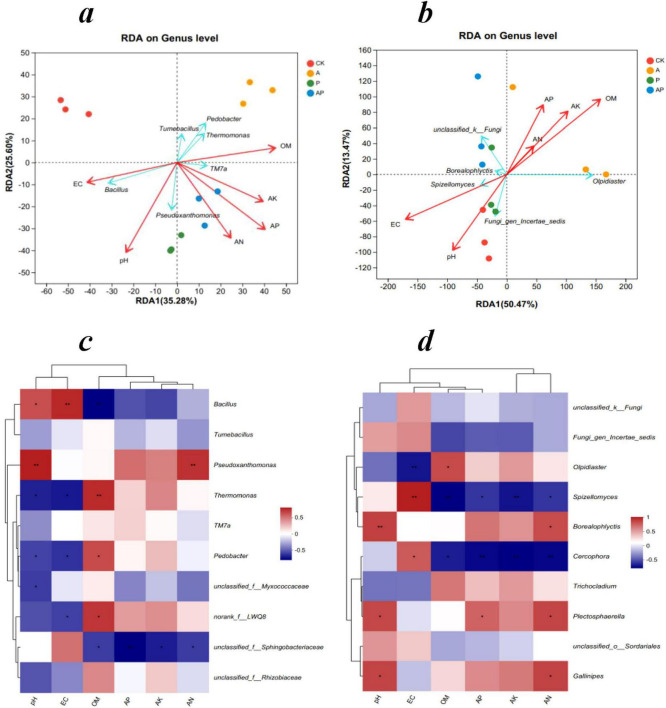
Redundancy and Pearson correlation analyses of microbial communities at the genus level and soil variables. Redundancy analysis of bacterial **(a)** and fungal **(b)** communities with soil variables, and Pearson correlation analysis of bacterial **(c)** and fungal **(d)** communities and soil variables. *P < 0.05; **P < 0.01. CK, control treatment; A, 90 mg/LN-ethylglycine acid; P, 90 mg/L Catechol; and AP, mixed treatment. OM, organic matter; AN, alkali-hydrolyzed nitrogen; AP, available phosphorus; and AK, available potassium.

Soil microbial diversity is important in maintaining soil nutrient cycling and soil ecosystem sustainability and is therefore often used as an indicator of soil health (Blay et al., 2017; Wang et al., 2020). Bacterial RDA results showed that these two axes explained 60.88% of the total variance. *Pedobacter* species showed highly significant negative correlation with EC and pH and highly significant positive correlation with OM. *Pseudoxanthomonas* species showed highly significant positive correlation with EC, pH, AN, AP, and AK and highly significant negative correlation with OM. The *TM7a* species showed a highly significant negative correlation with EC and pH and a highly significant positive correlation with OM, AN, AP, and AK (Figure 5a). This is consistent with previous studies showing that an increase in bacterial abundance, particularly that which promotes plant growth, can stimulate nutrient uptake and growth (Lu et al., 2015; Zhou et al., 2023). Fungal RDA showed that these two axes explained 75.29% of the fungal variation. *Olpidiaster* species showed highly significant negative correlation with EC and pH and highly significant positive correlation with OM, AN, AP, and AK. *Borealophlyctis* and *Spizellomyces* species showed highly significant positive correlations with EC and pH. The highly significant positive correlations with OM, AN, AP, and AK were highly significantly negatively correlated (Figure 5b). Previous studies have indicated that pH is a key factor influencing soil microbial diversity (Lanzén et al., 2015), and soil nutrient conditions (e.g., soil carbon and nitrogen) are important factors that limit the growth of microorganisms in the soil (Zhao et al., 2019). The present study confirmed the findings of previous studies that organic acids and phenolics in WV, particularly high concentrations of organic acids and phenolics, increased the effectiveness of soil nutrients and favored the growth of soil microorganisms (Zhou et al., 2022).

Pearson correlation analysis of bacterial communities with soil characteristics showed that the relative abundance of *Pseudoxanthomonas* was significantly and positively correlated with pH and AN, and the relative abundance of *Pedobacter* was significantly and negatively correlated with pH and EC, and significantly and positively correlated with OM (Figure 5c). Pearson’s correlation analysis of fungal communities with soil factors showed that the relative abundance of *Olpidiaster* was highly significantly negatively correlated with EC and significantly positively correlated with OM. *Spizellomyces* was highly significantly positively correlated with EC and highly significantly negatively correlated with OM and AK. The relative abundance of *Borealophlyctis* was highly and positively correlated with pH and significantly positively correlated with AN (Figure 5d).

### 3.7 Microbial community network analysis

**FIGURE 6:**
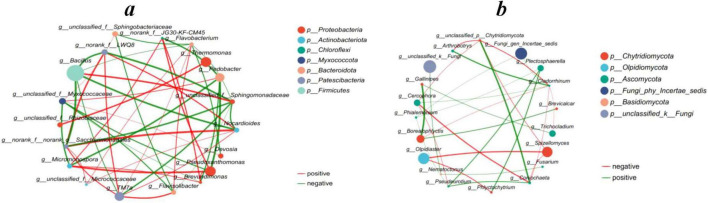
Network-based analysis: **(a)** single factor correlation network of bacterial community, **(b)** single factor correlation network of fungal community. The top 20 species with total abundance at the classification level were selected, and the correlation coefficient was ≥ 0.6, *P* < 0.05.

Spearman’s correlation analysis was performed on the abundance of species in each treatment to create a one-way correlation network which analyzed species-to-species relationships and illustrated the interactions among species in each treatment sample. Network analysis showed that *Pseudoxanthomonas* species of *Proteobacteria* were negatively correlated with *Pedobacter* species of *Bacteroidota* (Figure 6a). Fungal network analysis showed that *Olpidiaster* species of *Olpidiomycota* were positively correlated with *Spizellomyces* species of *Chytridiomycota* (Figure 6b).

### 3.8 Functional predictive analysis of microbial communities

**FIGURE 7:**
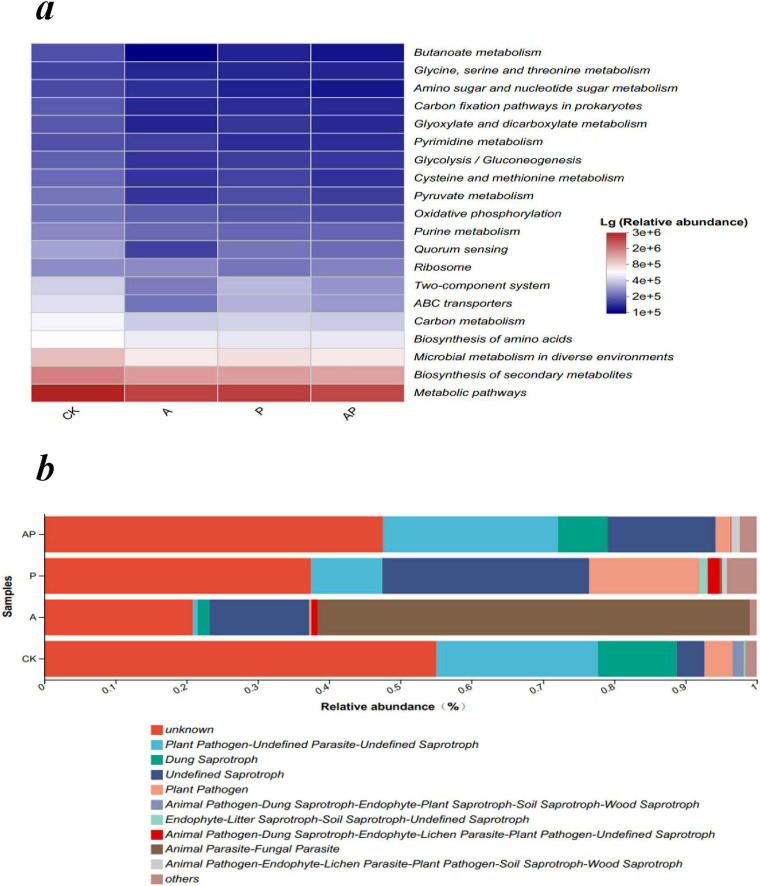
Function prediction of microbial community. **(a)** The main pathways on the KEGG level 3 of the bacterial community. **(b)** FUNGuild functional category abundance of fungal community. CK, control treatment; A, 90 mg/LN-ethylglycine acid; P, 90 mg/L Catechol; and AP, mixed treatment.

PICRUSt was used to estimate the functional attributes of bacterial communities (Wang et al., 2024). The results of the bacterial community KEGG level-3 pathway analysis showed that compared with the control, N-Ethylglycine acid (A), Catechol (P), and their combined application (AP) decreased the functional abundance of pathways related to the biosynthesis of secondary metabolites, metabolism in various environments, amino acid biosynthesis, carbon metabolism, ABC transporters, and the two-component system (Figure 7a). In soil ecosystems, microorganisms produce diverse secondary metabolites that facilitate communication, competition, and interactions with other organisms and the environment (Crits-Christoph et al., 2018).

FUNGuild is a technique used by ecological societies to categorize and analyze fungi in a simple and reliable way to classify large libraries of fungal sequences into ecologically essential classes. Fungi are classified into three major groups based on their nutritional strategies: pathogenic, saprophytic, and symbiotic (Tian et al., 2024). The four treatments exhibited the functional abundance of Plant Pathogen-Undefined Parasite-Undefined Saprotroph in the order CK > AP > P > A, and the functional abundance of Dung Saprotroph in the order CK > AP > A > P (Figure 7b). This suggests that N-ethylglycine (A) and Catechol (P) in WV decreased the functional groups of pathogenic fungi and the saprotroph population, whereas the mixture of N-ethylglycine acid and Catechol (AP) inhibited the reduction effect and reduced the stability of the soil microflora community. In conclusion, the mixture of acids and phenols in WV can increase the number of functional groups of soil pathogenic fungi and saprophytic bacteria, interfere with the metabolic pathways or translocation processes of soil microbial communities, and reduce their metabolic rate or efficiency, thus weakening their overall effects. Specifically, the antagonistic effects of acids and phenolics on WV depend on their concentrations in plants, relative proportions, and specific mechanisms of their effects on plant physiological processes. Further experiments are required to gain a deeper understanding of their detailed mechanisms of action and biological effects.

## 4 Discussion and conclusion

Research findings indicate that the main acidic and phenolic substances in WV can promote the growth and development of tomatoes. Among them, Picolinic acid and Catechol exhibit particularly prominent growth-promoting effects. However, when acidic and phenolic substances are used in combination, most show an antagonistic effect. The underlying mechanisms are as follows. Firstly, acidic and phenolic substances have differential regulation of soil physical and chemical properties. Acidic substances significantly reduce the soil pH value, while phenolic substances significantly increase the pH value. Both can reduce the soil EC value and increase the nutrient content. The effects of the mixed treatment on pH and EC offset each other, causing the soil physical and chemical properties to approach those of the control, but the nutrient-enhancing effect is weaker than that of the single treatment. This phenomenon is directly related to the chemical properties of acidic and phenolic substances: acidic substances release soil-fixed nutrients (such as N, P) by dissociating H^+^, and phenolic substances enhance the nutrient adsorption capacity by adjusting the charge of soil colloids. In the mixed treatment, the regulation efficiency of the two on the soil micro-environment may be weakened due to acid—base neutralization. Secondly, it is closely related to the up-regulation of endogenous IAA, GA_3_, and CTK contents and the down-regulation of the ABA content. Phenolic substances exert their effects by stimulating hormone synthesis and enzyme activity, while acidic substances enhance plant vitality by disrupting the physical interactions of the cell wall and promoting cell acidification. However, the growth-promoting effect of the mixed treatment is significantly lower than that of the single treatment, possibly due to the chemical reaction combination (such as acid—base neutralization) or competitive adsorption between acidic and phenolic substances, resulting in a decrease in the effectiveness of bioactive components. Finally, acidic and phenolic substances drive the remodeling of the structure and function of the rhizosphere microbial community. Acidic and phenolic monomers exert growth-promoting effects by promoting the colonization of beneficial microorganisms (such as *Pedobacter, Pseudoxanthomonas*). *Pedobacter* improves soil ecology by degrading complex carbon sources and resisting environmental stress, while *Pseudoxanthomonas* is involved in nitrogen and phosphorus metabolism to enhance nutrient availability. However, the mixed treatment enriches the potential pathogen *Spizellomyces* and down-regulates functional pathways such as bacterial secondary metabolite synthesis and carbon metabolism, indicating that the acid-phenol antagonism may reduce the stability of the soil micro-ecosystem by activating the functional groups of pathogenic fungi and interfering with the microbial metabolic network. The research results reveal the growth-promoting application mechanism of WV components and promote the comprehensive and high-value utilization of biomass resources. In future agricultural applications, the separate application of acidic or phenolic substances can be used as a green growth promoter to promote crop growth by regulating hormone balance and soil micro-ecology. However, the mixed application of the two should be avoided to prevent the antagonistic effect from reducing the effectiveness. Future research can further analyze the interaction patterns of acidic and phenolic substances under different concentration ratios, screen synergistic growth-promoting combinations, and provide a theoretical basis for the precise application of WV.

## Data Availability

The original contributions presented in the study are included in the article/Supplementary material, further inquiries can be directed to the corresponding author.

## Footnotes

1
https://www.majorbio.com
