# Supplementary material for: New enlightenment on the regulatory effects of acids and phenolic compounds in wood vinegar, a by-product of biomass pyrolysis, on tomato production
Source: Front Microbiol. 2025 Jul 16;16:1538998. doi: 10.3389/fmicb.2025.1538998 (PMC12307434; doi:10.3389/fmicb.2025.1538998)
Supplement: Supplementary file 1 [file Table_1.docx]

**Table S1: Relative content of main compounds in WV**

| **WV** | **Compound** | **Formula** | **Peak time /min** | **Comparative content (%)** | **Concentration（****mg/L）** |
| --- | --- | --- | --- | --- | --- |
| **Acids** | N-Ethylglycine | C_4_H_9_NO_2_ | 12.42 | 18.46 | 15,510 |
|  | Lactic acid | C_3_H_6_O_3_ | 7.42 | 14.3 | 14,460 |
|  | 2-picolinic acid | C_6_H_5_NO_2_ | 11.09 | 10.25 | 9,540 |
|  | Palmitic acid | C_16_H_32_O_2_ | 19.32 | 4.9 | 4,730 |
|  | 2-ketoadipate | C_6_H_8_O_5_ | 10.05 | 3.46 | 3,378 |
|  | Glycolic acid | C_2_H_4_O_3_ | 7.68 | 2 | 1,963 |
|  | Stearic acid | C_18_H_36_O_2_ | 21.11 | 1.64 | 1,653 |
|  | Adipic acid | C_6_H_10_O_4_ | 13.38 | 1.37 | 1,543 |
|  | Others |  | —— | 0.9 |  |
| **Phenols** | Catechol | C_6_H_6_O_2_ | 11.06 | 13.14 | 14,500 |
|  | Guaiacol | C_7_H_8_O_2_ | 9.84 | 11.04 | 9,030 |
|  | M-cresol | C_7_H_8_O | 8.63 | 1.64 | 1,643 |
|  | O-cresol | C7H8O | 8.50 | 0.34 | 328 |
|  | Others |  | —— | 0.29 |  |
| **Ketones** | 1,3-Cyclohexanedione | C_6_H_8_O_2_ | 10.74 | 1.03 | 1160 |
|  | 1,2-Cyclohexanedione | C_6_H_8_O_2_ | 15.33 | 1.03 | 1124 |
|  | Others |  | —— | 0.41 |  |
| **Esters** | Methyl jasmonate | C_13_H_20_O_3_ | 16.17 | 1.19 | 1235 |
|  | D-erythronolactone | C_4_H_6_O_4_ | 12.47 | 0.1 | 84 |
|  | Others |  | —— | 0.04 |  |
| **Aldehydes** | Succinate semialdehyde | C_4_H_6_O_3_ | 9.20 | 3.25 | 3231 |
|  | Glutaraldehyde | C_5_H_8_O_2_ | 10.54 | 0.2 | 123 |
|  | 2,5-dihydroxybenzaldehyde | C_7_H_6_O_3_ | 15.94 | 0.17 | 99 |
|  | Butyraldehyde | C_4_H_8_O | 10.50 | 0.15 | 94 |
|  | Others |  | —— | 0.03 |  |
| **Alcohols** | Perillyl Alcohol | C_10_H_16_O | 12.21 | 1.96 | 115 |
|  | 3-Methylamino-1,2-propanediol | C_4_H_11_NO_2_ | 9.46 | 0.44 | 254 |
|  | (-)-Dihydrocarveol | C_10_H_18_O | 10.72 | 0.29 | 178 |
|  | 3-Methylbenzyl Alcohol | C_8_H_10_O | 10.00 | 0.13 | 84 |
|  | Acetol | C_3_H_6_O_2_ | 13.71 | 0.12 | 83 |
|  | Others |  | —— | 0.26 |  |
| **Unknown** | —— |  | —— | 5.66 |  |

**Table S2: Biological shape statistics for all treatments**

| **Treatments** | | **Plant height**  **(cm)** | **Stem diameter**  **(mm)** | **Leaf area**  **(cm^2^)** | **Biomass(g)** | |
| --- | --- | --- | --- | --- | --- | --- |
|  |  |  |  |  | **Aboveground ( dry weight )** | **Underground ( dry weight )** |
| CK | CK | 13.27 | 4.32 | 9.02 | 0.59 | 0.17 |
| A1 | A1-1 | 21.60 | 5.69 | 8.88 | 1.19 | 0.40 |
|  | A1-2 | 21.23 | 5.83 | 11.35 | 2.57 | 0.56 |
|  | A1-3 | 25.17 | 6.17 | 14.10 | 1.16 | 0.28 |
| A2 | A2-1 | 16.47 | 4.42 | 8.55 | 0.99 | 0.31 |
|  | A2-2 | 15.53 | 4.56 | 8.71 | 0.96 | 0.32 |
|  | A2-3 | 17.43 | 5.23 | 8.45 | 1.32 | 0.33 |
| A3 | A3-1 | 21.57 | 6.19 | 16.05 | 1.66 | 0.70 |
|  | A3-2 | 23.83 | 6.73 | 14.63 | 1.68 | 0.58 |
|  | A3-3 | 22.10 | 6.28 | 13.10 | 2.35 | 0.69 |
| P1 | P1-1 | 22.30 | 5.44 | 13.77 | 1.73 | 0.38 |
|  | P1-2 | 22.43 | 6.26 | 13.03 | 1.85 | 0.52 |
|  | P1-3 | 24.13 | 6.28 | 13.68 | 1.88 | 0.58 |
| P2 | P2-1 | 19.23 | 5.96 | 12.88 | 1.20 | 0.35 |
|  | P2-2 | 19.50 | 5.52 | 10.54 | 0.77 | 0.31 |
|  | P2-3 | 20.47 | 5.52 | 11.67 | 1.04 | 0.37 |
| AP1 | AP1-1 | 23.47 | 6.22 | 12.43 | 1.73 | 0.56 |
|  | AP1-2 | 20.30 | 6.07 | 13.15 | 1.27 | 0.31 |
|  | AP1-3 | 17.77 | 5.28 | 10.85 | 0.95 | 0.24 |
| AP2 | AP2-1 | 18.40 | 5.68 | 13.47 | 1.09 | 0.37 |
|  | AP2-2 | 21.37 | 6.15 | 14.76 | 1.70 | 0.42 |
|  | AP2-3 | 23.43 | 5.85 | 14.12 | 1.33 | 0.33 |
| AP3 | AP3-1 | 14.37 | 4.76 | 8.19 | 0.88 | 0.32 |
|  | AP3-2 | 18.60 | 5.18 | 10.90 | 1.07 | 0.43 |
|  | AP3-3 | 19.57 | 5.66 | 10.09 | 1.67 | 0.35 |
| AP4 | AP4-1 | 20.37 | 5.89 | 10.20 | 1.02 | 0.31 |
|  | AP4-2 | 21.80 | 5.69 | 11.21 | 1.33 | 0.36 |
|  | AP4-3 | 21.80 | 5.64 | 8.88 | 1.35 | 0.43 |
| AP5 | AP5-1 | 20.97 | 5.86 | 11.29 | 2.53 | 0.62 |
|  | AP5-2 | 19.87 | 5.50 | 8.45 | 1.47 | 0.19 |
|  | AP5-3 | 20.87 | 5.87 | 7.80 | 0.96 | 0.29 |
| AP6 | AP6-1 | 21.67 | 6.06 | 9.98 | 1.61 | 0.54 |
|  | AP6-2 | 23.60 | 5.67 | 11.27 | 1.47 | 0.50 |
|  | AP6-3 | 21.97 | 5.16 | 8.73 | 1.81 | 0.52 |

Note : The ' -1, -2, -3 ' of each treatment represented the concentration of 30, 60, 90 mg/L, respectively, and the value of each treatment was the average of 9 repeated values.

**Table S3:** **Statistics of soil physical and chemical properties of all treatments**

|  |  | **PH** | **EC(μS/cm)** | **OM(g/kg)** | **AP((mg/kg))** | **AK(mg/kg)** | **AN(mg/kg)** |
| --- | --- | --- | --- | --- | --- | --- | --- |
| CK | CK | 6.89 | 2798.67 | 27.49 | 6.79 | 391.33 | 230.92 |
| A1 | A1-1 | 7.20 | 656.67 | 41.28 | 24.82 | 716.00 | 270.32 |
|  | A1-2 | 7.03 | 2562.33 | 43.47 | 26.62 | 1071.00 | 276.15 |
|  | A1-3 | 7.07 | 2641.33 | 53.06 | 23.93 | 1066.00 | 276.15 |
| A2 | A2-1 | 7.33 | 1970.00 | 33.36 | 27.77 | 1125.00 | 275.57 |
|  | A2-2 | 6.86 | 2565.33 | 41.88 | 26.94 | 707.33 | 224.47 |
|  | A2-3 | 6.36 | 2346.33 | 35.70 | 16.68 | 371.67 | 266.82 |
| A3 | A3-1 | 7.23 | 2919.33 | 50.66 | 32.43 | 1158.00 | 328.65 |
|  | A3-2 | 7.40 | 1335.00 | 51.06 | 32.95 | 1057.67 | 271.25 |
|  | A3-3 | 7.17 | 2627.33 | 47.25 | 34.57 | 915.00 | 316.40 |
| P1 | P1-1 | 7.22 | 2086.00 | 41.48 | 30.29 | 185.33 | 318.27 |
|  | P1-2 | 7.13 | 2707.67 | 39.55 | 25.73 | 654.33 | 288.40 |
|  | P1-3 | 7.05 | 1512.33 | 42.54 | 30.40 | 1144.67 | 231.23 |
| P2 | P2-1 | 7.14 | 2697.67 | 54.69 | 31.29 | 915.67 | 268.57 |
|  | P2-2 | 7.21 | 2640.33 | 53.56 | 27.58 | 875.00 | 307.65 |
|  | P2-3 | 7.08 | 2629.67 | 57.06 | 33.78 | 1122.00 | 304.15 |
| AP1 | AP1-1 | 6.99 | 1520.33 | 45.96 | 27.65 | 989.33 | 294.82 |
|  | AP1-2 | 7.09 | 2674.33 | 52.58 | 29.17 | 728.00 | 286.65 |
|  | AP1-3 | 6.86 | 2616.67 | 33.24 | 19.39 | 654.33 | 255.15 |
| AP2 | AP2-1 | 7.09 | 2767.33 | 48.41 | 32.86 | 843.00 | 250.48 |
|  | AP2-2 | 7.34 | 2553.00 | 49.31 | 44.56 | 814.67 | 318.15 |
|  | AP2-3 | 7.52 | 2477.33 | 61.12 | 38.34 | 912.33 | 323.98 |
| AP3 | AP3-1 | 6.74 | 2998.00 | 46.26 | 21.12 | 1069.67 | 280.82 |
|  | AP3-2 | 7.11 | 2651.67 | 53.41 | 25.37 | 1048.67 | 301.82 |
|  | AP3-3 | 6.97 | 2874.67 | 37.42 | 29.06 | 890.33 | 224.82 |
| AP4 | AP4-1 | 6.48 | 2519.67 | 27.96 | 29.30 | 787.33 | 232.98 |
|  | AP4-2 | 7.20 | 2700.33 | 38.84 | 23.70 | 636.00 | 251.65 |
|  | AP4-3 | 7.02 | 2466.33 | 45.07 | 22.23 | 622.00 | 237.65 |
| AP5 | AP5-1 | 7.22 | 2691.00 | 51.01 | 21.11 | 835.67 | 320.48 |
|  | AP5-2 | 7.53 | 660.00 | 46.70 | 23.25 | 1184.33 | 290.15 |
|  | AP5-3 | 7.02 | 2378.33 | 38.08 | 21.00 | 332.67 | 262.15 |
| AP6 | AP6-1 | 6.34 | 2456.33 | 60.57 | 27.54 | 934.33 | 262.15 |
|  | AP6-2 | 7.24 | 2604.00 | 46.41 | 29.12 | 862.00 | 304.15 |
|  | AP6-3 | 6.57 | 2418.33 | 41.15 | 14.48 | 428.67 | 321.65 |

Note : The ' -1, -2, -3 ' of each treatment represented the concentration of 30, 60, 90 mg/L, respectively, and the value of each treatment was the average of 3 repeated values.
